# Alcoholic Liver Disease: Current Mechanistic Aspects with Focus on Their Clinical Relevance

**DOI:** 10.3390/biomedicines7030068

**Published:** 2019-09-05

**Authors:** Rolf Teschke

**Affiliations:** Department of Internal Medicine II, Division of Gastroenterology and Hepatology, Klinikum Hanau, D-63450 Hanau, Academic Teaching Hospital of the Medical Faculty, Goethe University Frankfurt/Main, Frankfurt/Main, Germany; rolf.teschke@gmx.de; Tel.: +49-6181/21859; Fax: +49-6181/2964211

**Keywords:** alcohol dehydrogenase (ADH), microsomal ethanol-oxidizing system (MEOS), cytochrome P450 2E1, alcohol metabolism, endotoxins, reactive oxygen species (ROS)

## Abstract

The spectrum of alcoholic liver disease (ALD) is broad and includes alcoholic fatty liver, alcoholic steatohepatitis, alcoholic hepatitis, alcoholic fibrosis, alcoholic cirrhosis, and alcoholic hepatocellular carcinoma, best explained as a five-hit sequelae of injurious steps. ALD is not primarily the result of malnutrition as assumed for many decades but due to the ingested alcohol and its metabolic consequences although malnutrition may marginally contribute to disease aggravation. Ethanol is metabolized in the liver to the heavily reactive acetaldehyde via the alcohol dehydrogenase (ADH) and the cytochrome P450 isoform 2E1 of the microsomal ethanol-oxidizing system (MEOS). The resulting disturbances modify not only the liver parenchymal cells but also non-parenchymal cells such as Kupffer cells (KCs), hepatic stellate cells (HSCs), and liver sinusoidal endothelial cells (LSECs). These are activated by acetaldehyde, reactive oxygen species (ROS), and endotoxins, which are produced from bacteria in the gut and reach the liver due to gut leakage. A variety of intrahepatic signaling pathways and innate or acquired immune reactions are under discussion contributing to the pathogenesis of ALD via the five injurious hits responsible for disease aggravation. As some of the mechanistic steps are based on studies with in vitro cell systems or animal models, respective proposals for humans may be considered as tentative. However, sufficient evidence is provided for clinical risk factors that include the amount of alcohol used daily for more than a decade, gender differences with higher susceptibility of women, genetic predisposition, and preexisting liver disease. In essence, efforts within the last years were devoted to shed more light in the pathogenesis of ALD, much has been achieved but issues remain to what extent results obtained from experimental studies can be transferred to humans.

## 1. Introduction

The global burden of alcoholic liver disease (ALD) including the various stages like alcoholic fatty liver (AFL), alcoholic steatohepatitis (ASH), alcoholic hepatitis (AH), alcoholic fibrosis (AF), alcoholic cirrhosis (AC), and alcoholic hepatocellular carcinoma (AHCC) is immense, based on the high disease frequency worldwide with variable occurrence from one country to the other [1,2,3,4,5,6,7]. This compares with the overall rare liver injury caused by industrial products including solvents like carbon tetrachloride [8,9,10], or potentially toxins such as synthetic drugs [11,12,13] and ingredients of herbal products including herbal traditional Chinese medicine (TCM) [12,13]. Among alcoholic liver injury and non-alcoholic liver injury many pathogenetic steps are unexpectedly quite similar despite chemical dissimilarities of the causing compounds.

Mechanistic steps whereby alcohol causes early stages of liver injury can well be studied in animal models [14,15,16,17], and their results have successfully been transferred to human alcoholic liver disease, as detailed in various review articles [18,19,20]. As a reminder, human alcoholic liver disease develops from voluntary intake of alcohol demanded mostly by patients with an alcohol problem, and it is also a nature-based disease because alcohol is produced from sugar containing fruits derived from nature via natural, human supervised fermentation using baker’s yeast. Alcohol represents a short chain chemical with the formula C_2_H_5_OH, whereby the 2 C atoms are derived from glucose.

The focus of the present article is on various mechanistic and risk factors of clinical relevance involved in the development of ALD, viewed as a complex disorder with a possible poor outcome. Pathogenetic results are mostly based on studies in animals or human cell cultures, which help unravel disease complexities.

## 2. Literature Search and Data Review

Published reports were systematically searched for in electronic databases of Medline (source PubMed) using the search terms: Alcoholic liver disease, alcoholic fatty liver disease, alcoholic steatohepatitis, alcoholic hepatitis, alcoholic cirrhosis, endotoxins, reactive oxygen species (ROS), alcohol metabolism, alcohol dehydrogenase (ADH), microsomal ethanol-oxidizing system (MEOS), cytochrome P450 2E1 (CYP 2E1), catalase, and mitochondrial acetaldehyde dehydrogenase (ALDH). Publications of the first 50 hits from each searched segment were analyzed. The search was completed on 22 July 2019. Prior to the final analysis, the publications were assessed regarding clinical quality and data completeness. The final selection of publications was restricted to those in English language to ensure transparent accessibility.

## 3. Alcohol and Acetaldehyde Metabolism

As expected, much emphasis has been paid on the pathogenetic role of alcohol and acetaldehyde metabolism for initiation and perpetuation of alcoholic liver disease [3,5,6,7,15,16,17,18,19,20]. Respective data were derived from clinical and experimental studies, which created occasionally refreshing and sometimes more critical discussions around few specific topics of interest [18,19,20]. Clearly, ALD is not primarily the result of malnutrition as assumed many decades ago but due to the ingested alcohol and its metabolic consequences although malnutrition may contribute to disease aggravation.

Unless otherwise stated, the terms of alcohol and ethanol are used interchangeably in the text. Clearly, blood alcohol levels are commonly the result of intestinal resorption after alcohol consumption, however, small amounts of alcohol are produced by intestinal bacteria from ingested sugar if consumed in high amounts or under conditions of bacterial overgrowth, and this may lead to measurable blood alcohol levels that are not relevant clinically or legally in the context of driving. This condition is known as auto-brewery syndrome [21,22].

### 3.1. Alcohol Metabolism

The human body is well prepared to get rid of the consumed alcohol in order to prevent its accumulation and more serious acute health problems. Alcohol removal proceeds preferentially through enzymatic degradation [20,23,24].

#### 3.1.1. Alcohol Dehydrogenase

After ingestion, alcohol is metabolized in small amounts by the alcohol dehydrogenase (ADH) of the gastric mucosa by a mechanism known as first pass metabolism [24,25,26,27]. In addition, the mucosa of the small intestine contains with the microsomal ethanol-oxidizing system (MEOS) another enzyme, capable of metabolizing ethanol in vitro but its quantitative importance for local metabolism in the intestinal tract has not been studied [28]. Most of the ingested alcohol is rapidly absorbed by the intestinal mucosa and reaches the liver via the portal vessel system, where the alcohol will partly remain or bypass the liver and enter the systemic circulation resulting in measurable blood alcohol levels during several hours after ingestion [11,29]. Little amounts leave the body in the urine or is exhaled [29], while some alcohol may be found in the fat tissue and initiate mediator-based interactions within the liver [30].

Within the liver, alcohol is metabolized principally by the two enzymes, ADH and the microsomal ethanol-oxidizing system (MEOS), with no significant role for peroxisomal catalase [20,31,32,33,34]. Hepatic ADH consists of multiple forms with differences in their properties [34], has been characterized in detail, and is well prepared to down break ethanol to acetaldehyde [20,34]. Despite being available in abundancy, hepatic ADH is confronted with several issues: ADH activity requires nicotine amide dinucleotide (NAD^+^) as cofactor and competes thereby with the hepatic ALDH that also requires this cofactor; the pH optimum in vitro of ADH activity is outside the physiological range; alcohol in high concentrations is poorly handled by hepatic ADH as evidenced by the low Michaelis–Menten constant for ethanol; and hepatic ADH activity is not upregulated during prolonged alcohol consumption, not helping remove large amounts of alcohol during a longer period. In more detail, maximum ADH activity in vitro is found at a pH 10.4 and thereby in the alkaline range, the Michaelis–Menten constant (*K*_M_) of 1 mM for ethanol signifies full saturation of ADH already at low alcohol concentrations with plateau formation of alcohol degradation at higher alcohol concentrations [20,34].

The ADH dependent metabolism of ethanol to the toxic acetaldehyde C_2_H_4_O and the concomitant production of NAD + H^+^ have a significant pathogenetic impact on the initiation of alcoholic fatty liver disease (AFL), the first stage of ALD [32,34]: (1) hepatic acetaldehyde is injurious to its mitochondria, impairs acetaldehyde oxidation, decreases fatty oxidation contributing to AFL, and facilitates the conversion of hydroxyproline to procollagen, the precursor of collagen as a key component of liver fibrosis and cirrhosis [32], while (2) excess of NAD + H^+^ promotes collagen formation and additionally increases α-glycerophosphate and facilitates triglyceride accumulation in the liver cell by trapping fatty acids, enhances lipogenesis by promoting fatty acid synthesis, and impairs the citric acid cycle [32,34]. Overall, these two conditions related to acetaldehyde and NAD + H^+^ are cornerstones of ALF and partially also of subsequent disease stages like liver fibrosis as the precursor of cirrhosis.

#### 3.1.2. Microsomal Ethanol-Oxidizing System

Although present also in the gastrointestinal tract [35,36,37,38,39,40,41], MEOS is predominantly found in the liver, when it was first described in 1968 [42] and 1970 [43]. Based on these early investigations and various other studies [42,43,44,45,46], and as summarized recently [20,23], MEOS can now be characterized as follows: the reaction converts ethanol to acetaldehyde, MEOS depends on reduced nicotinamide adenine dinucleotide phosphate (NADPH + H^+^) or a NADPH regenerating system, and requires molecular oxygen [31,42,43,44,45,46,47,48,49]; key components of MEOS are several form of cytochrome P450 (CYP) with preference of its CYP 2E1 isoform, the NADPH-dependent cytochrome P450 reductase, and phospholipids [45,47,50,51]; MEOS is most active at a physiological pH, has a Michaelis–Menten constant of 7–11 mM and thereby active at intermediate and high alcohol concentrations, and is inducible in its activity following prolonged alcohol consumption [42,43]. Apart from ethanol, other aliphatic alcohols and various chemicals are known substrates of this special enzyme system [20,23].

The pathogenetic role of MEOS for ALD is confined primarily to the produced acetaldehyde, which impairs mitochondrial functions and is involved in collagen formation, but it remains unclear whether the high production of NADP^+^ resulting from NADPH + H^+^ use via MEOS for ethanol oxidation contributes to the pathogenesis of ALD [32].

MEOS resides in the endoplasmic reticulum of the hepatocytes from which the microsomal fraction can be prepared by ultracentrifugation [42,43,44,45,46,47,48]. During the preparative procedures, the microsomal fraction becomes contaminated by cytosolic ADH and peroxisomal catalase, which is certainly not a membranous constituent of the microsomal membranes as occasionally claimed, as MEOS is clearly an enzyme system different from ADH and catalase [42,43,46,48] and was also successfully isolated from these other enzymes by column chromatography following solubilization of microsomal membranes [45,49] reproduced in other confirmative studies [52].

New stimulating aspects regarding the pathogenetic background of alcoholic liver disease focus on the circadian rhythms [53,54]. First of all, alcohol modifies the expression of genes responsible for the circadian clock and regulating metabolic pathways [53]. Through yet unclear mechanisms involving SIRT-1, alcohol-based disruption in circadian rhythms may contribute to the initiation and perpetuation of alcoholic liver injury. There is also good experimental evidence for circadian rhythms of hepatic ADH and MEOS activities [54]. In particular, the circadian peak in ADH activity fell near the time of maximum blood ethanol clearance rates both in groups of rats injected with a single ethanol dose and in rats continuously exposed to ethanol for 22 weeks. At all time-points, however, hepatic ADH activities remained at a lower level and fluctuated less in the chronic group compared with either the acute or control groups. Instead, MEOS activity levels showed a prominent rhythm that was opposite with 180 degrees out-of-phase with the ADH rhythm in the chronic group, associated with low MEOS activities and lack of variation over the circadian span. On theoretical grounds, circadian rhythm variations of hepatic enzymes oxidizing ethanol could influence pathogenetic steps in alcoholic liver disease.

### 3.2. Overall Alcohol Metabolism

The degradation of the consumed alcohol proceeds via gastric ADH, hepatic ADH, and hepatic MEOS [55]. Under normal conditions, hepatic ADH is likely responsible for a major part of the ethanol metabolism, whereas hepatic MEOS could account for 20% to 25% of the alcohol metabolism in vivo [33]. However, the contribution of MEOS in alcohol metabolism will be increased at higher alcohol levels and following chronic alcohol use. Its high *K*_m_ value for ethanol favors the role of MEOS at higher alcohol concentrations, and induction of MEOS by chronic alcohol use removes alcohol more quickly under conditions of preexisting and long-lasting alcohol consumption. It has also been suggested that when corrected for microsomal losses during preparation, half to two thirds of the increase in the rate of ethanol oxidation after chronic alcohol use can be accounted for by MEOS [33]. Conservatively speaking and considering variable results, MEOS may contribute >25% of overall hepatic alcohol metabolism at high alcohol concentrations and after prolonged alcohol abuse considering mostly kinetic characteristics and also, rarely, inhibitory studies [55], with details outlined recently [23].

Metabolic aspects of alcohol with associated redox changes and production of the hepatotoxic acetaldehyde are closely related to the pathogenesis of ALD [34,55].

### 3.3. Acetaldehyde Dehydrogenase

Produced from ethanol via ADH and MEOS, acetaldehyde is oxidized by hepatic ALDH to acetate, which in turn leaves the liver and is further oxidized in various extrahepatic organs [32,34,56,57]. Hepatic ALDH consists of several isoenzymes and is present in various compartments of the liver cell [34]. Similar to the alcohol oxidation by ADH, the ALDH reaction requires NAD^+^, which is reduced to NADH + H^+^, with the consequence that both enzymes contribute to the decreased cellular NAD^+^/NADH + H^+^ ratio [34,55]. This has a major impact on hepatic intermediary pathways, which require NAD^+^ or are inhibited by excess NAD + H^+^ [34]. Certainly, part of the redox changes can be attenuated by reducing equivalents in the form of NADPH + H^+^ required for ethanol oxidation via MEOS [23].

For the pathogenesis of ALD most important is the finding that prolonged ingestion of alcohol leads a reduced capacity of liver mitochondria to metabolize the acetaldehyde generated by ADH and MEOS [56]. The impaired mitochondrial ALDH activity causes a vicious cycle with increased levels of acetaldehyde with its toxicity in various organs including the liver, where toxicity is due to the highly reactive molecule and its covalent binding to subcellular membranes and enzymes [32,56].

## 4. Reactive Oxygen Species

For understanding the pathogenesis of ALD the occurrence and action of ROS is of pivotal importance [7,23,58,59,60,61]. Along the NADPH dependent MEOS reaction also involving CYP 2E1, acetaldehyde and a variety of ROS are generated from incomplete split of molecular oxygen [7,23]: ethoxy radical CH_3_CH_2_O^•^, hydroxyethyl radical CH_3_C(**^•^**)HOH, acetyl radical CH_3_CHO^•^, singlet radical ^1^O_2,_ superoxide radical HO^•^_2_, hydrogen peroxide H_2_O_2_, hydroxyl radical HO^•^, alkoxyl radical RO^•^, and peroxyl radical ROO^•^. ROS undergoes covalent bonding to macromolecules within the liver cells including those present in membranes of subcellular organelles like mitochondria with their structural proteins and phospholipids. These injurious effects at the molecular and subcellular level evoke the transition of AFL to ASH and AH, as outlined in detail previously [7,58,59,60,61].

## 5. Gut–Liver Axis

Largely neglected although known from studies in Germany published since the middle of the eighties [62,63,64,65,66], new interest emerged on the role of endotoxins and the intestinal microbiome for the start and perpetuation of alcoholic liver disease [19,67,68,69] with analysis of the enzymes involved in metabolizing ethanol [28] and localized in various segments of the gastrointestinal tract [28,35,36,37,38,39,40,41]. More specifically: (1) The gastrointestinal tract of animals exhibits MEOS activity shown to be upregulated due to chronic alcohol consumption [35,36,37,38] with MEOS activities present in the stomach [36], small intestine [35,36], colon [36,37,38], as well as rectum [36]. This suggests that NADPH-cytochrome P450 reductase and phospholipids are also present in the gastrointestinal mucosa, otherwise MEOS would not function, an assumption based on studies in the liver [23,28,67] whereby the reductase, phospholipids, and CYP are essential components of hepatic MEOS [23,28,32,46,48,51,52,69]. (2) Since ADH is also found in the gastrointestinal tract together with MEOS [36], both enzymes present in the same mucosal cell may exert joint actions through exchange of reducing equivalents and speed up the metabolism of ethanol to its highly toxic acetaldehyde [28] in a way similar to the liver cell [20]. (3) The intestinal MEOS modifies via intestinal CYP 2E1 microbiome conditions and endotoxin generation [28,67], an important aspect not considered in some early reports [62,63,64]. (4) There is co-existence of MEOS with its constituent CYP 2E1 in the small intestine and colon [39]. (5) Additional immunochemical studies on the localization of the ethanol-inducible CYP 2E1 in the rat alimentary tract revealed the occurrence of immunoreactive CYP 2E1 only in the duodenal and jejunal villous cells but not in the ileum or distal colon of animals receiving the control diet [39]. (6) After ethanol treatment, however, CYP 2E1 was now expressed also in the proximal colon, associated with an increased CYP 2E1 content in the duodenum and jejunum as compared to animals receiving the control diet [39]. (7) Under clinical aspects of interest, CYP 2E1 is found in the colon of humans, with higher mRNA levels of CYP 2E1 in the descending colon and the sigmoid colon as compared to the ascending colon [41]. (8) Although data on CYP 2E1 in the gastrointestinal tract are lacking for patients with an alcohol problem respective, an upregulation can be assumed resulting in ROS generation [7,20], whereby some ROS will be needed for the function of MEOS [20]. (9) Since intestinal CYP 2E1 is part of the intestinal microbiome it may through ROS production contribute to the initiation of ALD [20,67].

**Figure 1:**
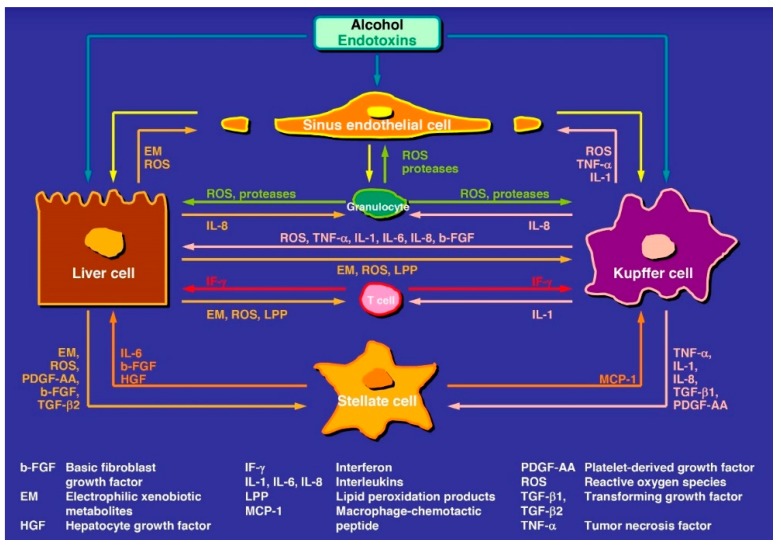
Tentative steps leading to early and intermediate stages of alcoholic liver disease. The pathogenesis involves various mediators and cell types of the liver, some of the steps need confirmation and are therefore hypothetical. The original figure was published in a recent report [7] and is reproduced with permission of the publisher Taylor and Francis (Didcot, UK).

Regarding pathogenesis, good evidence now exists that products called endotoxins are produced by intestinal bacteria and injure the intestinal mucosa, causing leakage of the intestinal mucosa barrier, allowing an increased uptake of endotoxins from the intestinal tract and release into the liver where they trigger liver injury (Figure 1) [7,19,28,67,68,69].

## 6. Hepatic Active Mediators and Signaling Pathways in ALD

The abundancy of publications on various pathogenetic aspects in ALD is impressive shown with few selected review articles, original reports, and associated mechanistic pathways presented with variable working models and concepts in graphical abstracts [3,5,7,11,16,19,20,23,28,32,34,53,55,56,58,70,71,72,73,74,75,76,77]. Abundancy and variability are as expected, considering the variability of human ALD stages and data derived from rare human studies as compared to highly frequent experimental studies using animal models or in vitro cell techniques with partially contradictory and debated results difficult to transfer to human disease settings. In line with these considerations and restrictions, the conclusion has been reached that a satisfactory unifying mechanism for individual susceptibility, initiation, and progression of alcoholic liver injury is not available [77]. Under these aspects, the only constants are: (1) ethanol is metabolized in the liver cell and acetaldehyde is its first oxidation product; (2) these chemicals are targeting the liver cells (LCs) and all non-parenchymal cells (NPLCs) (Figure 1) such as Kupffer cells (KCs), hepatic stellate cells (HSCs), liver sinusoidal endothelial cells (LSECs), as well as intrahepatic granulocytes, lymphocytes, and monocytes; and (3) the various stages of ALD.

### 6.1. Alcoholic Fatty Liver

According to the five-hit working hypothesis with a tentative cascade of events, ALF is the result of the first injurious hit [20] and characterized by accumulation of lipids within the LCs due to intracellular metabolic and structural alterations [3,18,55,61] rather than triggered by active mediators derived from NPLCs [61,78], although few extrahepatic mechanisms contribute to the development of AFL [78]. However, NPLCs may play an increasing pathogenetic role if simple AFL progresses to the more injurious ASH with LCs, which still contain some fat, or to AH (Figure 1) [7].

### 6.2. Alcoholic Steatohepatitis and Alcoholic Hepatitis

ASH may result from the second injurious hit and AH from the third one [20]. Both represent progressing stages of ALD with more severe clinical features of AH as compared to ASH, triggered by various mediators such as interferons, interleukins, tumor necrosis factor, and various growth factors that have been released from NPLCs like Kupffer cells and stellate cells or liver cells (Figure 1) [20,78,79]. Another focus is on molecules of the danger-associated molecular patterns or death-associated molecular patterns summarized as DAMPs, which represent altered metabolism products of necrotic or stressed cells and are deemed as alarm signals by the innate immune system [3,78,80,81]. In a vicious loop, inflammatory agents are DAMP generators, and DAMPs create a pro-inflammatory state. These conditions are closely associated with immune mechanisms of both innate and acquired immunity triggering perpetuation of ALD [82].

### 6.3. Alcoholic Fibrosis and Cirrhosis

The fourth hit is dominated by increased collagen formation and other extracellular matrix proteins, triggered by activated HSCs, and leading to AF and irreversible AC [20,70]. This is commonly observed as transition from ASH or AH, although it occurs rarely also without these two intermediate stages. On a molecular basis, acetaldehyde and lactate but not ethanol stimulate collagen synthesis in hepatic fibroblasts [83], conditions leading to AF and AC.

### 6.4. Alcoholic Hepatocellular Carcinoma

In rare cases, a fifth hit initiates the development of AHCC, mostly occurring in patients with cirrhosis. This final hit scenario of carcinogenesis is triggered by acetaldehyde and ROS through the generation of DNA adducts, which promote mutagenesis, and interference with methylation, synthesis, and repair of DNA [3,84,85], suggested as a possible contributing role of SIRT-1 [20]. These overall events will enhance AHCC susceptibility, keeping in mind that ethanol itself is not a carcinogenic chemical.

## 7. Risk Factors

On theoretical grounds, assessing risk factors of ALD is best achieved using a prospective study protocol and evaluating disease stages with liver histology obtained by liver biopsy, an invasive procedure. In praxis, however, such a prospective study is not feasible for several reasons including ethical considerations, risks associated with the invasive diagnostic approach, the requirements of a large study cohort, and the overall missing therapeutic consequences for patients with ALD. Therefore, with a few exceptions only results from retrospective studies can be used with all its limitations when risk or modifying factors of ALD are to be discussed [3,5,7,19,20,70,78,86,87,88,89,90]. For some potential risk factors, published results are variable, partially contradictory, or obtained in small study cohorts, not allowing firm final conclusions. Selected risk factors of potential clinical relevance and based on sufficient evidence are discussed.

### 7.1. Amount of Consumed Alcohol

Good evidence exist that the amount of daily alcohol consumption is a major risk factor for ALD, and this applies preferentially to advanced stages such as AC [3,86,87,88,89] including AC patients with ascites [87,88]. When calculated in g absolute alcohol consumed daily, intake of 40 to 80 g by males and of 20 to 40 g by females for 10 to 12 years is a common risk factor of severe ALD, with preference of ASH, AF, and AC [78,89].

### 7.2. Gender

Female alcoholic patients are at a higher risk for ALD as compared to alcoholic men [3,88,89]. In particular, women have more advanced liver disease at time of diagnosis, experience a more severe clinical course within a shorter time of alcohol abuse, and consume less alcohol compared to men [89], in line with a lower thresholds for development of alcoholic liver injury [88,89]. This gender difference can be traced back to higher blood alcohol concentrations in woman compared to men who consume the same amount of alcohol, resulting from a lower proportion of body water in females than in males of equal body weight [78] and from a lower ADH-dependent first pass metabolism in the gastric mucosa [26]. Under discussion are also gender based differences in the sensitivity of hepatic KCs to endotoxins generated in the gut [78].

### 7.3. Genetic Predisposition

Genetic and epigenetic factors have to be implicated because only a minority of alcoholic patients suffer from advanced ALD like AC, and monozygotic twins show a higher concordance rate for AC as compared to dizygotic twins [3,78]. Under discussion are genetic modifications of alcohol metabolism that may facilitate progression to more severe stages of ALD [3,78], with special reference to polymorphism in ADH and ALDH genes [17]. Genome wide associations studies have identified specific genetic markers also in genes encoding cytokines and antioxidant enzymes that are related to the progression of ALD [78,90].

### 7.4. Alcohol-Unrelated Liver Disease

Preexisting liver diseases based for instance on infections by hepatitis B or C viruses are major risk factors of ALD and disease progression [3,78], whereas the role of other chronic liver diseases like hemochromatosis or α_1_-antitrypsin deficiency is less clear [3]. Obesity and associated nonalcoholic steatohepatitis are also known risk factors of ALD progression [3,78].

## 8. Conclusions

In order to simplify the understanding of pathogenetic steps leading to the various stages of ALD, a proposal of five-hit sequelae is presented based on various mechanistic considerations. These include (1) the hepatic metabolism of ethanol to the heavily reactive acetaldehyde; (2) the metabolism proceeds via the alcohol dehydrogenase (ADH) and the cytochrome P450 isoform 2E1 of the microsomal ethanol-oxidizing system (MEOS); (3) the resulting metabolic disturbances modify not only the liver parenchymal cells but also non-parenchymal cells like Kupffer cells, hepatic stellate cells, and liver sinusoidal endothelial cells; (4) these cells are activated by acetaldehyde, reactive oxygen species (ROS) generated during ethanol metabolism via MEOS, and endotoxins, which are part of the liver–gut axis, being produced from intestinal bacteria and reaching the liver due to gut leakage; (5) most importantly, reactive acetaldehyde and ROS covalently bind to cellular proteins and phospholipids initiating and perpetuating liver injury; (6) various intrahepatic signaling pathways involving mediators like interferons, interleukins, and growth factors govern injurious and activating effects on a variety of cellular targets; and (7) innate or acquired immune reactions are under discussion contributing to the pathogenesis of ALD. These mechanistic steps are partially derived from results of experimental studies, therefore to be viewed as tentative, and may not necessarily transferable to humans with ALD. Evidence is better for clinical risk factors like the amount of alcohol used daily for more than a decade, gender differences with higher susceptibility of women, genetic predisposition, and preexisting liver disease. Expanding future studies on the issues of pathogenesis may help providing new therapy options in addition to current approaches of strict alcohol abstinence.
